# COVID-19 and beyond: the ethical challenges of resetting health services during and after public health emergencies

**DOI:** 10.1136/medethics-2020-106965

**Published:** 2020-10-16

**Authors:** Paul Baines, Heather Draper, Anna Chiumento, Sara Fovargue, Lucy Frith

**Affiliations:** 1 Division of Health Science, Warwick Medical School, University of Warwick, Coventry, UK; 2 Department of Primary Care and Mental Health, University of Liverpool, Liverpool, UK; 3 Law School, Lancaster University, Lancaster, UK; 4 Institute of Population Health, University of Liverpool, Liverpool, UK

**Keywords:** ethics, public health ethics, public policy

COVID-19 continues to dominate 2020 and is likely to be a feature of our lives for some time to come. Given this, how should health systems respond ethically to the *persistent* challenges of responding to the ongoing impact of the pandemic? Relatedly, what ethical values should underpin the resetting of health services after the initial wave, knowing that local spikes and further waves now seem inevitable? In this editorial, we outline some of the ethical challenges confronting those running health services as they try to resume non-COVID-related services, and the downstream ethical implications these have for healthcare professionals’ day-to-day decision making. This is a phase of recovery, resumption and renewal; a form of reset for health services.^1^ This reset phase will define the ‘new normal' for healthcare delivery, and it offers an opportunity to reimagine and change services for the better. There are difficulties, however, healthcare systems are already weakened by austerity and the first wave of COVID-19 and remain under stress as the pandemic continues. The reset period is operating alongside, rather than at the end, of the pandemic and this creates difficult ethical choices.

## Ethical challenges of reset

### Balancing the greater good with individual care

Pandemics—and public health emergencies more generally—reinforce approaches to ethics that emphasise or derive from the interests of communities, rather than those grounded in the claims of the autonomous individual. The response has been to draw on more public health focused ethics, ‘if demand outstrips the ability to deliver to existing standards, more strictly utilitarian considerations will have to be applied, and decisions about how to meet the individual's need will give way to decisions about how to maximise overall benefit’.^2^ Alongside this, effective control of pandemics requires that we all adopt strategies to reduce disease transmission such as the lockdown measures instituted by governments worldwide. Individual liberties are curtailed for the greater good.

Together, these factors shift the weighting of ethical concepts to emphasise the individual *within* a community.^3 4^ For many years, public health ethicists and practitioners have drawn attention to the importance of the health of the whole community^5^ and the broader determinants of health, including the built environment and the way that society is structured.^6 7^ Public health emergencies, such as COVID-19, demonstrate our mutual dependencies and highlight the need to prioritise the interests of the community. The difficulty of balancing these tensions between the interests of the ‘wider community’ and the patient as the ‘first concern’ has been well rehearsed. In the reset period, how to further the public good is contested; should health services prioritise the response to COVID-19; or should we now be trying to give equal or greater priority to providing non-COVID services? It has been argued that the response to COVID-19 will produce much greater detrimental effects on population health than the disease itself, including the impact of those who need healthcare for non-COVID conditions not receiving treatment.^8 9^ Thus, in the current pandemic, how to promote the public good is by no means clear and which wider community’s interests should be prioritised needs careful ethical consideration.

Attention also needs to be paid to relationships between healthcare professionals and patients, as elements of non-verbal communication are inhibited by wearing masks; the calming and reassuring gesture of touch is prohibited or distorted by the use of personal protective equipment (PPE); and patients have to attend appointments on their own without any support, no matter how difficult or traumatic the consultation is expected to be.^10^ This raises important ethical questions about how the demands of infection control should be balanced against the need for personalised, dignified and supportive care. Responding to these competing demands can result in moral distress for healthcare professionals who feel ill-prepared or unable to pursue ethically appropriate actions.^11^ COVID-19 has created new and uncertain circumstances that continue to disrupt our understandings of what ‘good care’ looks like and, in so doing, shifts the underpinning values or assumptions on which care is based, raising new ethical considerations for day-to-day decision making.

### Resource allocation

Resource allocation is a perennial problem in health systems and the persistence of COVID-19 will magnify concerns about National Health Service (NHS) resources long after the first wave. With the suspension of many non-Covid services from March 2020 in the UK, the backlog of demand for non-Covid services has grown, and the pressures on healthcare services are even greater. At the same time, healthcare is necessarily less efficient because of COVID-19 infection control precautions. Each healthcare interaction takes longer because of the time it takes to clean equipment and the treatment area, don and doff PPE, and patients cannot be left waiting in shared rooms but must be tightly scheduled.

In the first wave of the pandemic, the analysis focused on resource allocation *between* patients with COVID-19.^12^ In this reset period, attention must now turn to how to allocate resources between those with COVID-19 and all other patients, including those whose conditions are not life-threatening and these kinds of decisions need focused ethical scrutiny.

## What should be done?

Guidance on ethical responses for the acute phase of a pandemic is readily available.^13^ This is not the case when considering how health systems *ought* to reset in the immediate aftermath of a pandemic or other public health emergency. We are at a juncture where the challenges brought on by the response to COVID-19 are forcing the re-evaluation of traditional clinical ethical approaches. The theoretical basis is shifting to give greater weight to the interests of the community as a whole. For example, the principle of justice may need to be given greater prominence, as well as a more self-conscious and widespread inclusion of values such as solidarity and reciprocity in decision making at both individual and organisational levels.^14^

The pandemic has also highlighted how longstanding health, housing, financial and racial inequalities interact with the COVID-19 virus, exacting a disproportionate impact on those already facing disadvantage and discrimination.^15^ In the healthcare context, an additional dimension to this is the disproportionate impact of COVID-19 on healthcare workers from Black, Asian and minority ethnic communities.^16^ As Richard Horton has argued, COVID-19 is not a pandemic it is a syndemic. Seeing Covid as a syndemic directs the focus towards the social and biological interactions that increase someone’s susceptibility to worse health outcomes.^17^ Consequently, in the reset phase, ethical decision making must pay more attention to the interaction between COVID-19 and longstanding health and socioeconomic inequalities.

The speed of response necessary for the first wave of the COVID-19 pandemic meant that decisions were made with little public scrutiny or consultation.^18^ But this approach *cannot* be justified in the reset period. The statutory, and ethical, obligation to maintain public involvement in decisions relating to service provision was reiterated by NHS England in March 2020.^19^ And this obligation extends to the scrutiny of the ethical values and arguments that underpin—implicitly or explicitly—the ways that services are reconfigured and the decisions about which patients and staff will bear the costs of reconfiguration.

The transition through repeated waves of COVID-19, while not just re-establishing but also resetting NHS services, will require new ways of thinking about how to integrate public health, organisational and systems-based approaches *with* clinical ethics. All health systems need to think about which ethical considerations are important in the reset period, which values and interests should take precedence, and how competing interests can and should be managed. These matters deserve more explicit consideration in ethical and practitioner literature and much wider public consultation.
